# Benchtop NMR signal enhancement of metabolites in urine extract using SABRE

**DOI:** 10.1039/d5cc06186e

**Published:** 2026-01-09

**Authors:** Simon Fleischer, Jing Yang, Kerti Ausmees, Indrek Reile, Neil MacKinnon, Jan Gerrit Korvink, Sören Lehmkuhl

**Affiliations:** a Institute of Microstructure Technology, Karlsruhe Institute of Technology Hermann-von-Helmholtz-Platz 1 76344 Eggenstein-Leopoldshafen Germany simon.fleischer@kit.edu soeren.lehmkuhl@kit.edu; b National Institute of Chemical Physics and Biophysics Akadeemia tee 23 12618 Tallinn Estonia

## Abstract

Metabolites in a urine extract are signal-enhanced by SABRE††SABRE: Signal Amplification By Reversible Exchange. hyperpolarization and detected using a benchtop NMR spectrometer. Quantification by standard addition is demonstrated for endogenic urinary nicotinamide (vitamin B3). Even higher sensitivity is achieved in an automated setup for multi-scan SABRE experiments. This hyperpolarization scheme is able to expedite biomarker detection and quantification, while maintaining low infrastructure requirements.

SABRE: Signal Amplification By Reversible Exchange.

## Abstract 2

Enhancing NMR signals of urinary metabolites using SABRE hyperpolarization in an automated benchtop-scale setup.

## Graphical abstract

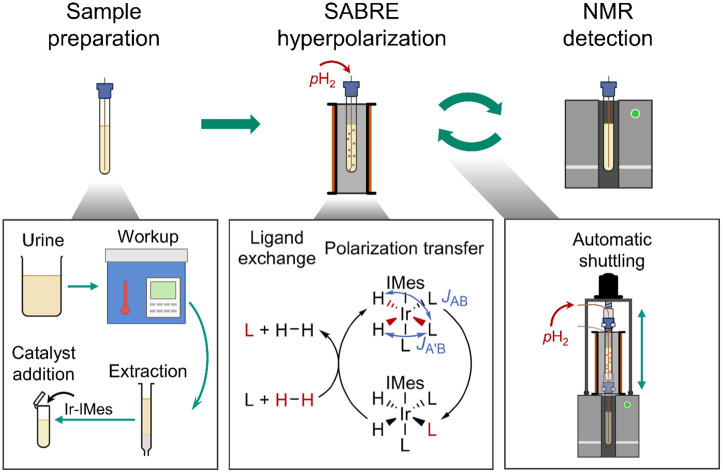

Rapid analysis of body fluids has become an important focus in health and metabolomics research. Urine is of particular interest, as it can be sampled non-invasively and can give a comprehensive record of an individual's metabolic state.^1,2^ The Human Metabolome Database lists over 3000 endogenous low-molecular weight metabolites in urine, only a fraction of which have been quantified.^2,3^ A range of these metabolites can in principle be detected by NMR, but the low natural concentration of many analytes poses a significant challenge. This is a particular hindrance for benchtop NMR spectrometers, which could offer an affordable way for routine analysis.

In this work, we increase the concentration sensitivity of benchtop NMR for select metabolites in a urinary extract by Signal Amplification By Reversible Exchange (SABRE) hyperpolarization.^4^ In contrast to superconducting high-field NMR instruments, benchtop spectrometers require less space, do not require cryogen refills, and their lower magnetic fields eases their integration into less specialized environments while adhering to safety regulations. These lower fields limit both sensitivity in non-hyperpolarized experiments, as well as chemical shift resolution. SABRE generates high polarization levels independent from the magnetic detection field, and allows for multiple polarization cycles and signal averaging, making it particularly attractive for overcoming the lower sensitivity of compact, low-field spectrometers (see Fig. 1).

**Fig. 1:**
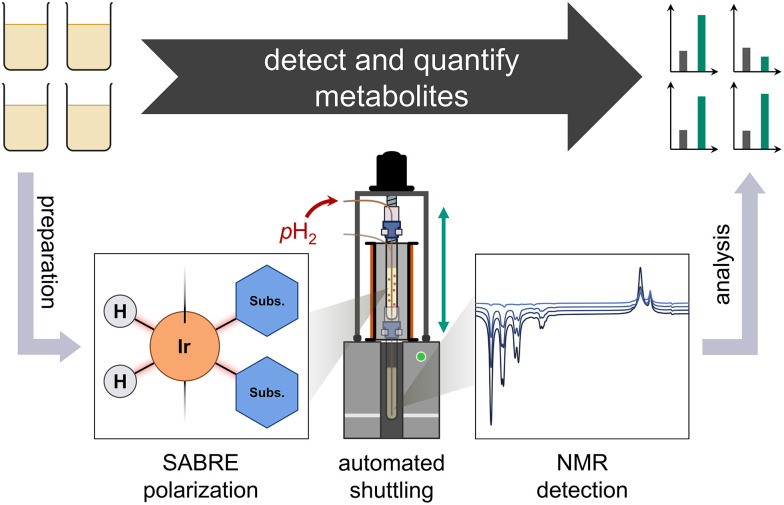
General detection and quantification procedure. Urine is processed, and relevant metabolites are extracted (see main text). Hyperpolarization using SABRE enhances select NMR resonances, which facilitates identification and quantification of metabolites, in conjunction with an automatic shuttling setup.

Over the past decades, several other techniques have been developed to hyperpolarize mixtures as well, including variants of dynamic nuclear polarization (DNP)^5^ and ParaHydrogen-induced polarization (PHIP).^6^ Dissolution-DNP was used to identify patients with chronic kidney disease,^7^ as well as in monitoring metabolic conversions, both in cells^8^ and *ex vivo*.^9^ PHIP was used in several biological studies, *e.g.* PHIP side arm hydrogenation for spectroscopic imaging of the conversion of pyruvate to lactate in transgenic mice.^10^ To our knowledge, no PHIP-based routine was reported in natural extract analysis.

The different hyperpolarization variants have specific advantages and disadvantages. Dissolution-DNP is a powerful hyperpolarization technique, but requires radicals to be added to the sample, as well as microwave irradiation at cryogenic temperatures, is limited to single-shot experiments, and often requires considerable preparation times.^11,12^ Overhauser-DNP is less experimentally demanding and allows for re-hyperpolarization,^13^ but requires small sample volumes of a few µL or less for *in situ* polarization, respectively a sub-second shuttling setup for polarization outside of the spectrometer.^14^ Hydrogenative PHIP allows for large enhancements, but necessarily employs hydrogenation, which makes it inherently single-shot and reduces the scope of target substrates.^15^ In contrast to that, non-hydrogenative PHIP (nh-PHIP) allows for re-hyperpolarization at a repetition rate similar to regular NMR, and indirect detection of various compounds binding to a transition metal complex.^16,17^ It has been used for detecting nicotine and its breakdown products in urine,^18^ and was recently proposed as a broad-scope method for urine metabolome analysis.^19^ However, hyperpolarized nh-PHIP signals represent analytes bound to the metal complex, with their distinct chemical shifts. Such signals need to be assigned to different species, which is not intuitive and complicates analysis.

SABRE hyperpolarization, in turn, allows for repeatable, direct polarization of target molecules. Polarization transfer is facilitated by the same class of catalyst as nh-PHIP, and yields hyperpolarized signals at the familiar chemical shifts of the analytes.^20,21^ However, SABRE hyperpolarization for natural mixture analysis has not yet been published to our knowledge. In this work, we employ a three-step process adapted from Reile *et al.*^22^ for urine analysis: urine extract preparation, SABRE hyperpolarization and NMR detection, as illustrated in Fig. 2.

**Fig. 2:**
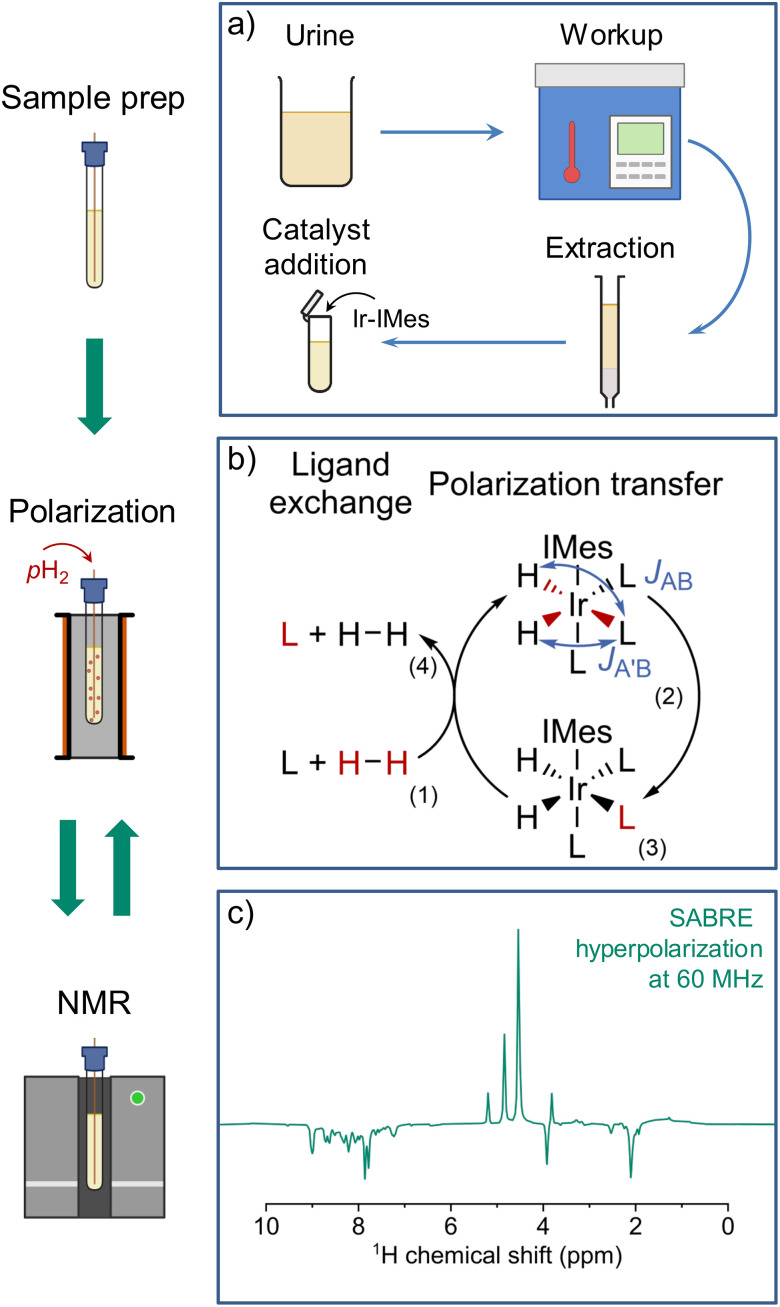
Full experimental procedure for urine extract hyperpolarization with SABRE. (a) The sample is prepared by autoclaving urine, which is centrifuged afterwards. The supernatant is collected and pH adjusted, before being loaded onto a solid phase extraction column. After drying, the analytes are eluted using deuterated methanol, and catalyst precursor is added to the resulting solution. (b) Parahydrogen gas is bubbled through the sample, facilitating SABRE catalyst activation. Polarization is transferred to analyte molecules (L) at a magnetic field of 6.5 mT. Starting from (1), spin alignment is transferred to the analyte *via* transient *J*-couplings (2), resulting in hyperpolarized analyte (3), which can be released into solution (4). (c) ^1^H SABRE spectrum acquired after polarization. Signals from hyperpolarized analytes can be identified by their negative amplitude. After spectrum acquisition, the sample can be returned to the transfer field and repolarized, as in (b).

In the preparation step (Fig. 2a), urine is first autoclaved to eliminate potential pathogens. After centrifugation and pH adjustment, a solid-phase extraction (SPE) adapted from published methods,^18,22^ yields a methanolic solution containing urinary metabolites. These metabolites are in roughly six-fold higher concentration than in the initial urine (see SI for details). SPE reduces the complexity of the metabolic mixture, easing identification on benchtop spectrometers with limited frequency resolution, and provides ideal conditions for SABRE hyperpolarization (see SI). While extraction is not strictly required, raw urine samples would still need to be stripped of unwanted substances, including salts, urea and ammonia, as shown in studies employing nh-PHIP.^16,23,24^

For the hyperpolarization step (Fig. 2b), the sample is SABRE-hyperpolarized at a magnetic field of 6.5 mT (experimental details see SI) by bubbling 99% enriched parahydrogen (pH_2_). Applying a magnetic field of 6.5 mT allows for optimum spontaneous polarization transfer to ^1^H on a broad range of organic compounds,^25^ particularly for N-heterocycles, including nicotinamide and its derivatives.^26^

In the NMR detection step (Fig. 2c), the sample is transferred to a 1.5 T benchtop spectrometer, using a linear shuttling setup (Fig. 3a) based on a previously published design, which facilitates sample transfer by a robotic arm.^27^ Linear shuttling allows for shorter transfer times and higher positional accuracy when moving between polarization transfer field and spectrometer. This ensures consistent polarization build-up and transfer timings, as well as a defined transfer path through the magnetic field, contributing to highly repeatable scan-to-scan performance (experimental details see SI). Consequently, a hyperpolarized ^1^H NMR spectrum of the urine extract is acquired, displaying enhanced resonances, which stem from endogenous urinary SABRE-compatible analytes.

**Fig. 3:**
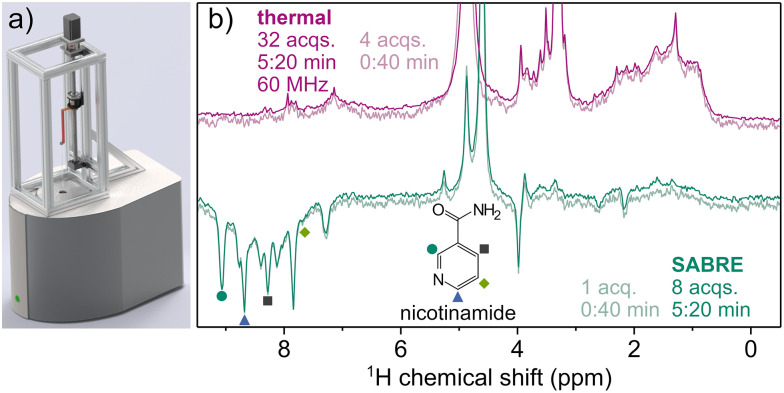
Shuttling setup employed for multiple acquisitions (a) and signal averaged spectra (b). (a) The shuttling setup consists of a stepper motor, which turns a threaded shaft, moving the shuttling head in its track. The NMR vial mounted within the shuttling head is connected to tubes maintaining a constant pressure of pH_2_. Bubbling can be switched on and off using an automatic bypass valve. To facilitate signal averaging, the sample is shuttled between the spectrometer for signal acquisition and an electromagnet generating a field of 6.5 mT for efficient SABRE polarization transfer. (b) Spectra after hyperpolarization (green) and reference spectra with thermal equilibrium polarization (purple) are shown, resulting from experiments with the number of scans chosen to maintain a similar total acquisition time. A single-acquisition SABRE spectrum and a reference spectrum after a similarly long acquisition time are overlaid for comparison. Hyperpolarized signals of nicotinamide are marked with symbols corresponding to their respective ^1^H nuclei.

The resulting hyperpolarized spectrum of urine extract is compared to a thermal reference spectrum recorded at 1.5 T in Fig. 3b). In the SABRE spectrum, the signals of non-hyperpolarized compounds are suppressed compared to the spectrum recorded using thermal equilibrium polarization, as the sample spends only 0.7 s at the detection field before acquisition, allowing for a limited build-up of field-derived polarization. As a result, the SABRE spectrum is significantly less crowded than the spectrum acquired at thermal equilibrium, especially in the range of 0.6–4.8 ppm. In contrast to this, the aromatic region (7–9 ppm) contains various hyperpolarized peaks.

These enhanced signals can be identified by their natural chemical shifts, with assignments verifiable by standard addition. We demonstrate this for nicotinamide (NAM), a form of vitamin B3, which is known to be particularly amenable to SABRE hyperpolarization.^28^ The presence of NAM was further confirmed, and its concentration estimated, by nh-PHIP (see SI).^29^ We note that the NAM resonances at 7.6, 8.3 and 8.7 ppm marked in Fig. 3b) overlap with other signals not stemming from NAM. These additional peaks are not baseline-resolved, hindering quantification of NAM based on the respective resonances. A slightly narrower linewidth can be achieved by further optimizing the hydrogen delivery system (details see SI).

In the experiments shown here, the NAM resonance at 9.1 ppm is well isolated and appears to be mostly background-free.

This allows us to quantify NAM in the initial urine by adopting the internal standard spiking approach that has been previously demonstrated for SABRE^28^ and nh-PHIP,^22,29^ to a methanolic urine extract (Fig. 4a). The observed relation between added NAM and increase in hyperpolarized ^1^H signal integrals follows a linear trend (Fig. 4b), despite the complex nature of sample composition and SABRE polarization transfer, which proceeds over several competing equilibrium states with individual rate constants.^30^ We applied linear regression analysis for all four resonances to determine the concentration of NAM in the extract from each resonance (inset in Fig. 4b).^28^

**Fig. 4:**
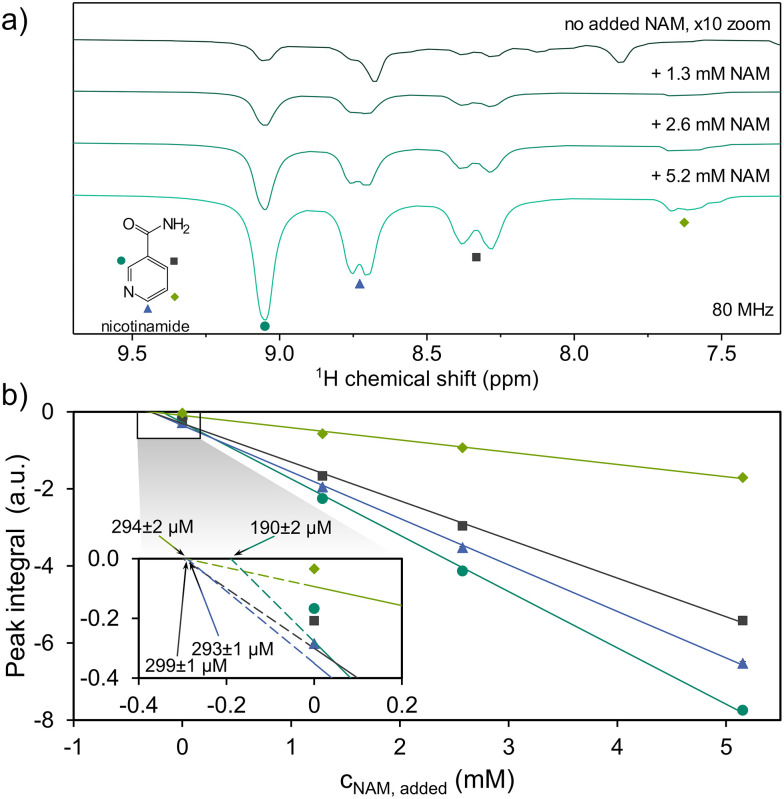
Spectra (8 scans each, recorded at 80 MHz) showing hyperpolarized peaks of nicotinamide (NAM) at different concentrations spiked into the extract sample (a), and concentration-integral plot of the peaks (b), all identified by the same color and symbol assigned to NAM ring protons shown in (a). The spectrum of extract without added NAM in (a) is magnified 10-fold to aid visibility. Extrapolating the regression lines in the inset of (b) permits the estimation of initial concentration of NAM in the extract sample. Three of the four peaks assigned to NAM are in good accordance. Despite this, the concentration of 190 ± 2 µM appears most plausible. See text for discussion.

Following the NAM resonance integral at 9.1 ppm (dark green circles), we arrive at a concentration of 190 ± 2 µM NAM in the employed sample. For the other resonances, we obtain higher initial concentrations of 294 ± 2 µM (7.6 ppm, bright green diamonds), 293 ± 1 µM (8.3 ppm, blue triangles) and 299 ± 1 µM (8.7 ppm, grey squares), respectively. This difference is attributed to the non-NAM signals found in the chemical shift range of 7.2–8.8 ppm, which cause an offset in integral value for these resonances.

Considering the dilution of extract (400 µL in a sample of 650 µL) and the six-fold concentration increase during extraction, assuming full recovery,^22^ we arrive at a concentration of 51 µM NAM in the initial urine. This value aligns with the 40 µM estimated by nh-PHIP (details see SI). The limit of quantization in a single scan is equivalent to 1.5 µM NAM in the initial urine. While low, the found value of 51 µM NAM is much higher than the reported normal urinary concentration of roughly 4 µM using HPLC.^31^ This discrepancy can be traced back to the workup procedure. In contrast to other studies, including work based on nh-PHIP,^18,22^ the urine samples in this work were sterilized by autoclaving, which can result in thermal degradation of larger NAM-containing molecules, such as nicotinamide adenine dinucleotide, releasing additional free NAM.^32,33^

SABRE experiments are often carried out with additional co-substrates to ensure an overabundance of coordination partners, which helps to stabilize the active species and fine-tune the exchange rate constants.^21,22,28,34^ Here, no co-substrates are used. While the individual metabolites are sub-stochiometric with respect to the SABRE catalyst, the sheer number of coordination partners in the urine extract stabilizes the catalyst complex in solution and prevents deterioration over the course of multiple acquisitions. This observation highlights the unexpected robustness and reversible ligand-exchange behavior at the Ir-catalyst in an environment of highly competitive substrate binding, providing insight that could be relevant to other fields, such as in the study of reaction mixtures and catalytic processes. Fitting resonances can improve peak separation in benchtop spectra,^35^ and increasing chemical shift resolution or applying more advanced pulse schemes, such as pure shift NMR,^36^ or 2D sequences, as routinely done in nh-PHIP routines,^17,37^ will aid in identifying and quantifying additional constituents.

Our procedure enables rapid detection of metabolic markers in a complex sample matrix, employing an affordable benchtop spectrometer. The chemoselective nature of SABRE, along with the extraction step, makes the technique useful for targeted analysis.^38^ After initial calibration by standard addition, conclusive ^1^H NMR spectra may be produced from just a single scan, requiring less than a minute. This allows adoption in high-throughput laboratories and in clinical settings near point-of-care, requiring fast, targeted identification and quantification of metabolites. While the related nh-PHIP procedure yields more hyperpolarized signals for the same extract (SI), the conceptual simplicity of SABRE and its ability to yield NMR data at the natural chemical shifts of analytes makes it the preferred choice as long as the analyte is amendable to SABRE. Considering that the known list of SABRE hyperpolarizable and metabolically relevant compounds already includes several valuable metabolites^39,40^ and drugs,^41,42^ the utility of SABRE in biochemical analysis is improving quickly.

Conceptualization: SL; data curation: SF, KA, IR; formal analysis: SF, KA, IR, NM, SL; funding acquisition: SL, IR, JK; investigation: SF, KA; methodology: SF, JY, IR; project administration: SL; resources: IR, NM, SL, JK; software: SF, JY; supervision: SL, JK; validation: SF, KA; visualization: SF, JY, KA; writing – original draft: SF; writing – review & editing: JY, KA, IR, NM, JK, SL.

## Conflicts of interest

There are no conflicts to declare.

## Supplementary Material

CC-062-D5CC06186E-s001

## Data Availability

NMR data for this article is available at RADAR4KIT at https://doi.org/10.35097/qfe8pn1s6cc9uzam. Supplementary information: additional experimental details and spectra. See DOI: https://doi.org/10.1039/d5cc06186e.
