# Supplementary material for: Benchtop NMR signal enhancement of metabolites in urine extract using SABRE
Source: Chem Commun (Camb). 2026 Jan 9;62(10):3342–6. doi: 10.1039/d5cc06186e (PMC12810685; doi:10.1039/d5cc06186e)
Supplement: CC-062-D5CC06186E-s001 [file CC-062-D5CC06186E-s001.pdf]

Supplementary Information (SI) for ChemComm.
This journal is © The Royal Society of Chemistry 2026

Benchtop Signal Enhancement of Metabolites in Urine Extract using SABRE

Simon Fleischer, Jing Yang, Kerti Ausmees, Indrek Reile, Neil MacKinnon, Jan Gerrit Korvink, Sören Lehmkuhl

Table of Contents

Urine workup routine 3
Solid phase extraction and SABRE sample preparation 3
Automated shuttling and acquisition on benchtop.. 4
NMR data acquisition and processing 6
Reproducibility using the shuttling setup.. 6
Chemical shift resolution 7
High-field characterization of extract and comparison with lyophilized urine 8
Urine extract investigation by nh-PHIP 11
References..13

Urine workup routine

Urine was collected from a self-reported healthy male volunteer from the morning midstream over the course of four days, resulting in a total volume of 950 mL. Each urine donation was frozen until workup. The volunteer's diet was not monitored or controlled, and he continued to report as healthy for 14 days following the donation. Informed consent was obtained from the participant. Urine sample handling was approved by the Ethics Committee of KIT (number A2024-003).

Urine samples were thawed and consolidated before sterilization. Sterilization was carried out in a Laboklav ECO 80M (SHP Steriltechnik, Germany) autoclave over 20 mins at 121 °C. Afterwards, the urine was centrifuged at 3220 G over 5 min (4000 RPM in Eppendorf Centrifuge 5810 with rotor A-4-61, Eppendorf, Germany). The supernatant was carefully pipetted off, split into aliquots and stored at -20 °C until further use.

Solid phase extraction and SABRE sample preparation

Frozen urine aliquots were thawed, and their pH-value adjusted to 7.34, using aqueous NaOH (6 M) and HCl (5 M) solutions. During thawing, some precipitate was observed, which fully dissolved upon reaching room temperature.

For solid phase extraction, Oasis HLB cartridges (6 cc/200 mg, 30 µm pores, Waters Corp., USA) were used. For the entire procedure, a mild vacuum was applied via a vacuum manifold, maintaining a flow rate of approx. 1 drop of liquid per second in each of the six cartridges used in parallel. Each cartridge was conditioned with 5 mL methanol and immediately afterwards rinsed with 5 mL of a 20 mM phosphate buffer solution adjusted to pH 7.4. Once the buffer solution level reached the protective frit over the adsorbent layer, each cartridge was loaded with 5 mL of autoclaved urine, allowed to elute until the protective frit was almost dry, and rinsed with 2 mL of purified water. A brown ring near the top of the resin layer of the cartridge remained. Vacuum was increased to dry the cartridges with ambient air for 4 h. Finally, analytes were eluted by adding 1.2 mL of deuterated methanol (NMR spectronorm 99,8%, VWR International, Germany) to the cartridge. The first 0.8 mL of eluate per column were collected, ensuring near-complete analyte recovery.[1] Each eluate batch was split into aliquots and stored at -20 °C until further use.

SABRE catalyst precursor [Ir(IMes)(COD)]Cl was synthesized in-house according to previously published procedures.[2,3] The solid precursor was stored under argon atmosphere until use.

SABRE samples were prepared from 400 µL (336 mg) of urine extract, 100 µL (85 mg) of 6.5 mM [Ir(IMes)(COD)]Cl solution, and 150 µL (127 mg) of neat, degassed methanol-d_4. This yields a sample containing 1 mM iridium catalyst and extract diluted to 61.5%. For nicotinamide quantification, the added methanol was partially substituted by 6.5 mM NAM solution, with all other fractions kept constant.

The SABRE catalyst precursor was activated for 20 – 30 mins by applying a parahydrogen pressure of 7 bars and bubbling intermittently. This activation procedure followed the same shuttling routine as for the spectra acquisition described in the main text, to monitor the activation progress. Before beginning acquisition, a quick shim (x, y, z, z^2) was performed, to compensate for magnetic field inhomogeneities introduced by the bubbling capillary. Hydrogen atmosphere was maintained until the end of the respective experiment. All experiments were conducted using 99% $p\text{H}_2$, unless stated otherwise.

Automated shuttling and acquisition on benchtop

The linear shuttling system is constructed on an aluminum profile frame. It is comprised of a ball screw linear guide assembly (SFS1620-DM and 1620B-L400, Dold Mechatronics, Germany), a stepper motor (57SM110, Yingshi, China) controlled by a motor driver circuit (QY1249, Yingshi, China), a custom-built electromagnetic solenoid coil for generating milli-tesla-range magnetic fields for proton polarization transfer via SABRE, and a 3D-printed NMR tube holder. A 1.2 mm O.D. polyethylene capillary is used for gas delivery.

In addition, the bubbling process was automated by integrating a bypass shut-off valve with a servo motor, which was regulated by a microprocessor (Arduino UNO R3). The microprocessor and motor driver were integrated into a unified control unit. A mass flow controller (MC-500SCCM-D, Alicat Scientific, USA) was employed to regulate the gas flow rate during parahydrogen bubbling.

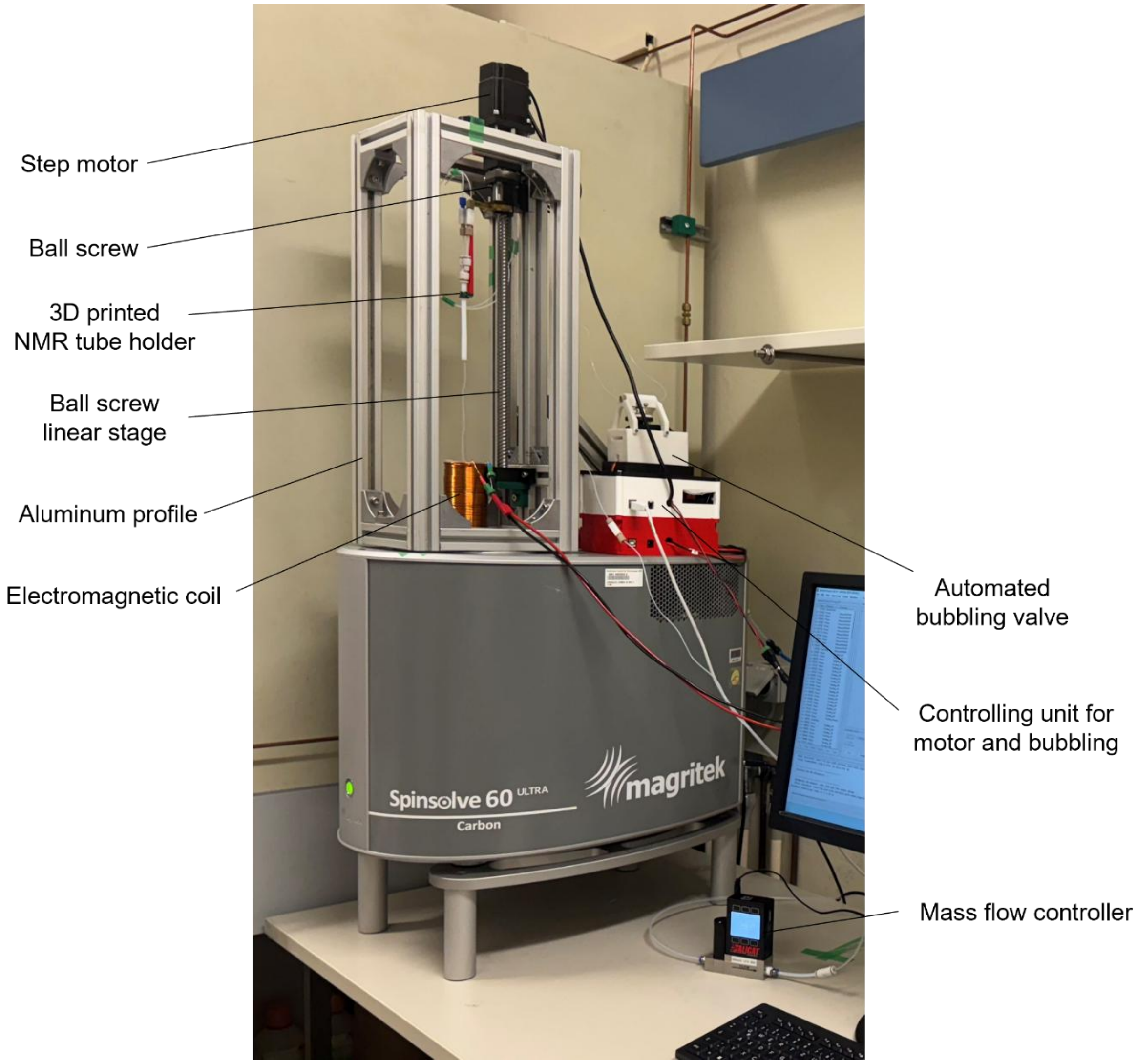

Figure S1: Photo of the automated shuttling system mounted on a 1.5 T benchtop NMR spectrometer (Spinsolve 60 Ultra), showing the shuttling stage, control electronics and hydrogen supply control. This setup was moved to a 2 T NMR spectrometer (Spinsolve 80) for the respective experiments.

A graphical user interface (GUI) was developed to control the stepper motor, servo valve, and experimental sequence (Figure S2a). The interface provides separate modules for motor control, bubbling control, and experiment control. In the motor control section, buttons allow the user to enable or disable the motor and to move the sample holder upward or downward. The bubbling control section regulates the status (ON or OFF) of the bubbling valve for SABRE pre-catalyst activation.

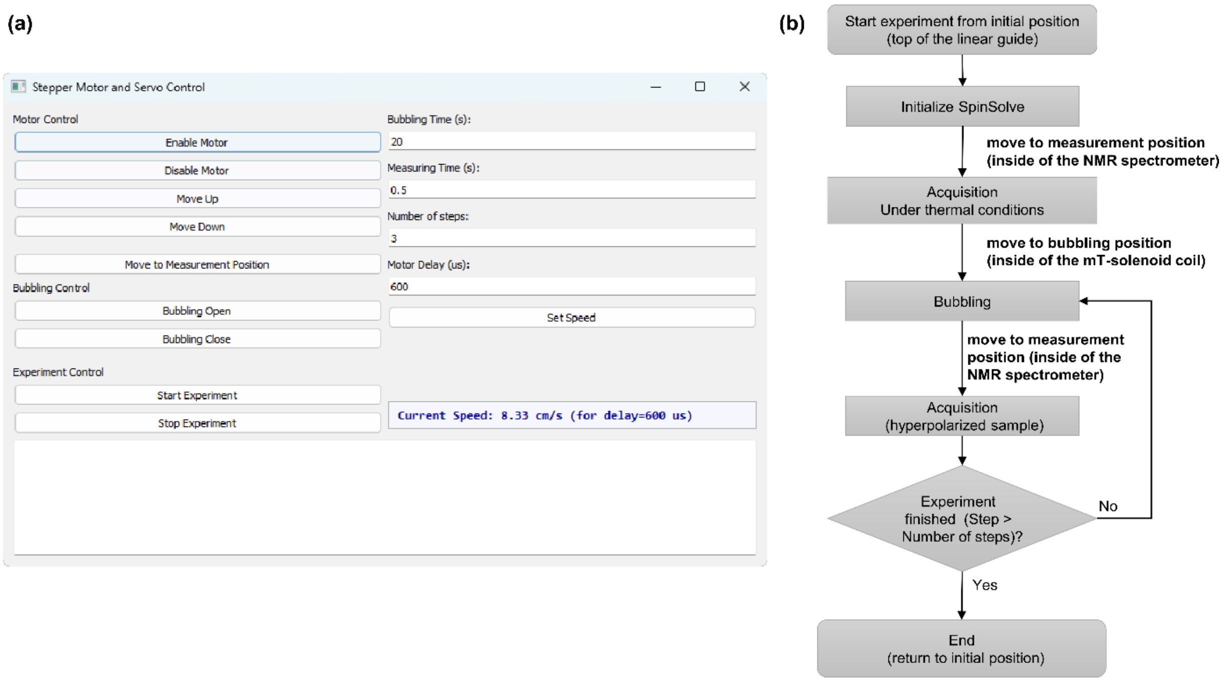

Figure S2: The designed GUI window (a) and default workflow chart of the automated shuttling system (b). The shuttling speed is automatically calculated based on the set motor delay, see below.

The experiment control section manages automated experimental sequences, including the start and stop of experiments, as well as timing parameters such as bubbling time, measuring time (delay between the sample arriving at the measurement position inside of the NMR spectrometer and signal acquisition), and motor delay for shuttling. The motor delay parameter defines the pulse interval for the stepper motor, thereby determining the translational velocity of the NMR tube during sample transfer. All parameters are adjustable through the GUI, enabling flexible and precise control of the automated shuttling and bubbling processes.

The translational motion of the sample tube in the linear shuttling system is governed by pulse control of the stepper motor. The motor advances by one step for each complete pulse cycle, consisting of both HIGH and LOW states. The shuttling velocity can be calculated from the screw lead and pulse timing according to

$$\text{Speed} = \frac{l}{n \cdot T} = \frac{20\ \text{mm/r}}{200\ \text{r}^{-1}} \cdot \frac{1}{2 \cdot 600\ \mu\text{s}} \approx 83.3\ \text{mm/s}, \qquad \text{(S1)}$$

where l is the lead of the ball screw determined by the screw pitch (20 mm for the SFS1620-DM ball screw), n is the number of steps per revolution, which is 200 steps for a 1.8° stepper motor, and T is the pulse period corresponding to one complete step cycle. Each step cycle consists of a HIGH and LOW signal, so the total period T equals twice the step delay. In this work, a default step delay of 600 μs was used, resulting in a translational velocity of approximately 83.3 mm/s.

The workflow of the automated shuttling and measurement sequence is illustrated in Figure S2b). The experiment begins with the sample positioned at the top of the linear guide, defined as the initial position. After system initialization, the control software establishes communication with the SpinSolve NMR spectrometer through its application programming interface (API). The sample is first shuttled downward into the NMR spectrometer without bubbling to acquire a reference spectrum under thermal conditions. Subsequently, the sample is shuttled upward into the solenoid coil, which generates a 6.5 mT polarization transfer field, where bubbling of parahydrogen is initiated for a predefined duration to achieve SABRE

hyperpolarization. Upon completion of the bubbling step, the stepper motor rapidly transfers the sample back to the measurement position. After the set delay has passed, the SpinSolve API is automatically invoked to initiate signal acquisition of the hyperpolarized sample. This process iterates according to the predefined number of steps until the experiment is completed, at which point the sample holder is returned to its initial position.

NMR data acquisition and processing

Spectra shown in Figures 2 and 3, main text, were recorded on a 1.5 T Magritek 60 Ultra spectrometer, using the default Proton sequence with a 90° pulse. Spectral width was 5000 Hz, with 16384 points acquired over 3277 ms.

Spectra shown in Figures 1 and 4, main text, were recorded on a 2 T Magritek 80 spectrometer, using the default Proton sequence with a 90° pulse. Spectral width was 5000 Hz, with 32768 points acquired over 6554 ms.

All benchtop data was acquired using SpinsolveExpert 2.02.14 and processed in MestReNova 14.1.2. Spectra were zero-filled to 65536 points. After applying an exponential apodization window of 0.70 Hz, spectra were referenced using the methanol singlet peak. After automatic baseline correction, using 3rd degree polynomials, phase adjustment was done manually, as automatic phasing performed poorly.

For signal averaging, multiple spectra were saved as a csv matrix, using the export script built into MNova, and loaded into a custom Python routine. For each spectral bin, this routine calculates the average value and outputs it to a new csv file, which can be loaded back into MestReNova for further analysis. For quantification, classical peak integration (“Sum” in MNova) was performed for each individual spectrum, reporting the average value.

Reproducibility using the shuttling setup

A demonstration of the reproducibility achieved by the shuttling setup is given in Figure S3 for 8 consecutive scans on a sample of solid phase urine extract. We note a systematic 9% increase in the methanol-d_4 signal at 4.87 ppm (Y5), caused by a slow loss of deuteration due to exchange with protium from parahydrogen gas. The generation of hyperpolarized hydrogen gas results in the high-intensity signal at 4.57 ppm (Y4), overlaid with the center resonance of HD, formed due to HD coupling of J = 45 Hz. Signals assigned to NAM (Y, Y1, Y2) show an increase in integral magnitude of up to 2% over the course of the experiment, likely caused by the accumulation of residual hyperpolarization from previous polarization cycles.

We note a higher linewidth than what is usually achieved on benchtop spectrometers of this type. Specifically, the linewidth of the signal at 9.1 ppm varies from 3.1 to 4.8 Hz over multiple experiments, which is in contrast to a linewidth of 0.3 Hz usually achieved. This is in large part attributed to the bubbling capillary, which enters the active volume of the spectrometer, and causes magnetic field distortions, which can only partially be compensated by shimming. In addition, the automatic shuttling process introduces minor deviations in the sample position, causing a minor fluctuation in linewidth as well.

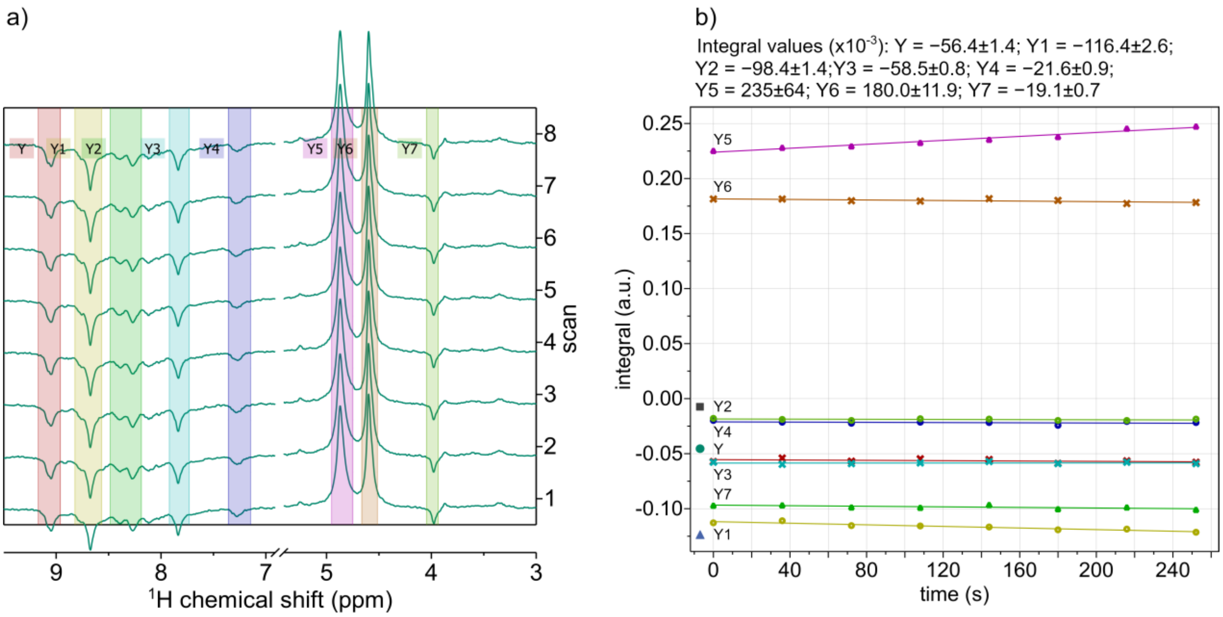

Figure S3: Spectra of 8 consecutive acquisitions using the automated shuttling setup (a) and corresponding integral-time plot (b) with accompanying integral values. Spectra were acquired in intervals of 36 s and processed as described in the text. Plot b) shows an increase in methanol singlet signal integral (Y5), attributed to slow protonation of the solvent. Other integrals remain consistent over the course of the experiment, with signals attributed to NAM (Y, Y1, Y2) varying by 2% or less.

Chemical shift resolution

In addition to the routine described above, we also measured single-shot spectra after long activation times using *p*H_2 with 50% enrichment, generated using a portable parahydrogen generator (HyperSpin Scientific, Germany) cooled by liquid nitrogen. The sample used here consisted of undiluted urine extract, 9 mM iridium catalyst and 1.5 mM TMSP-d_4 sodium salt. The catalyst was allowed to activate for 145 mins before acquisition. *p*H_2 was bubbled through the solution intermittently, and a pressure of 5.2 bars was maintained. Before acquisition, shimming of all gradients available on the Magritek 60 Ultra spectrometer was performed. The spectrum shown in Figure S4 was acquired using the default Proton sequence with a 90° pulse. Spectral width was 5000 Hz, with 32768 points acquired over 6554 ms.

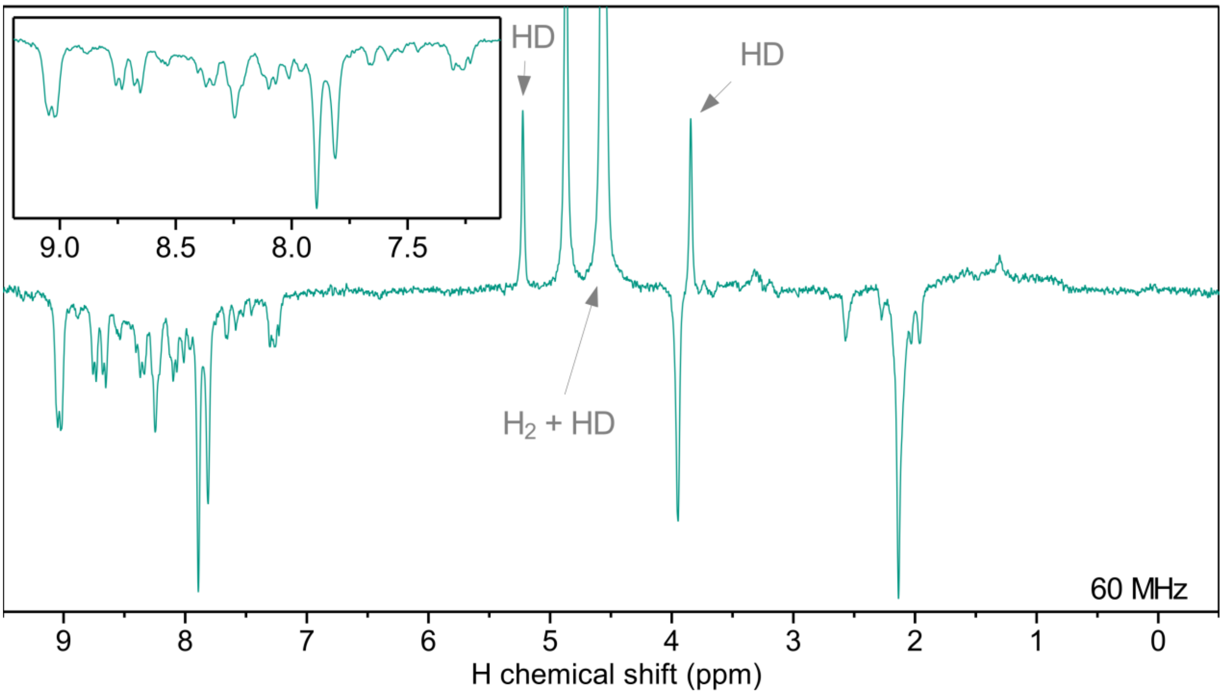

Figure S4: SABRE spectrum of urine extract after extensive activation and shimming, recorded in a single shot at 60 MHz. An exponential window of 0.3 Hz is applied. A zoomed-in view of the region from 7.1 – 9.2 ppm is provided in the inset. Signals stemming from hyperpolarized H_2 and HD gas are labelled.

After just a single 6.6 s acquisition, the resulting spectrum shows strong resonances in line with the spectra presented in Figures 2c) and 3b), main text. The reduced linewidth afforded by more extensive shimming further enhances the chemical shift resolution. Additional resonances in the NAM region are revealed, which are suspected to offset the concentration predicted when considering the signals at 7.6, 8.3 and 8.7 ppm.

This altered experimental procedure demonstrates the potential for high-throughput applications. Even though benchtop spectrometers tend to be less expensive than traditional room-scale devices, they no doubt remain the limiting factor regarding costs of a diagnostic SABRE setup. Sample activation, on the other hand, is the limiting step regarding throughput speed. Since activation does not need to proceed in the spectrometer, it can conceivably take place within an automatic processing line, with each sample only spending seconds in the spectrometer. Parallelization of the activation step would reasonably result in a throughput of multiple samples per minute.

Additionally, some hyperpolarized resonances will stem from analyte bound to the catalyst and not from analytes in solution (“free”). However, the free-to-bound ratio is rather reproducible for each analyte, as it is effectively governed by the combined concentrations of all other analytes. As long as this ratio is kept constant, a linear relationship of the SABRE signal and concentration is observed. Therefore, quantification is possible when calibrating for each compound individually as shown by Eshuis et al.[4]

High-field characterization of extract and comparison with lyophilized urine

To compare the composition of urine extract and treated urine, we also lyophilized urine. After autoclaving, the solids were separated by centrifuging, as above. Lyophilization of the supernatant over 48 h (Alpha 1-2 LDplus, Martin Christ Gefriertrocknungsanlagen GmbH, Germany) yielded a coarse, ochre powder, which was stored at -20°C until use. For ^{1}H NMR, 47 mg of the lyophilisate was dissolved in 700 µL of D_2O.

900 µL of urine extract was placed in a vacuum concentrator (Concentrator plus, Eppendorf SE, Germany) for 3.5 h at 30 °C. This yielded a dark brown sludge, which was dissolved in 700 µL D_2O.

Spectra for both solutions were acquired on a 500 MHz Avance NEO spectrometer (Bruker Biospin GmbH, Germany), using 64 scans. TMSP-d_4 sodium salt (3.4 mM for lyophilisate, respectively 4.6 mM for extract) was added as chemical shift reference. The resulting spectra are shown in Figure S5.

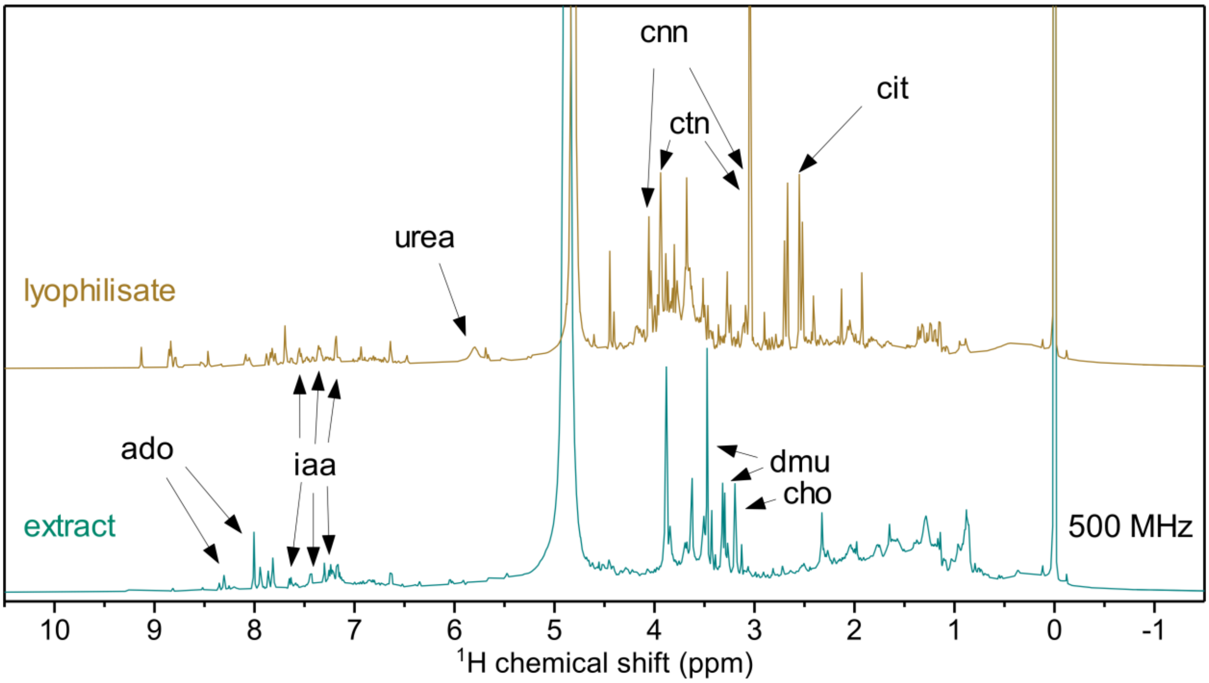

Figure S5: Spectra of redissolved lyophilized urine (ochre) and solid phase extract (green), both in D_2O, after 32 scans with an acquisition time of 5 s. Both spectra are referenced to the TMSP-peak at 0 ppm. Data was acquired with 125k points over a spectral width of 25 kHz. Spectra were processed in MestreNova: zero filled to 262144 points, exponential line broadening of 1.0 Hz, manually phase- and baseline-corrected using a 3rd order polynomial. Some signals are assigned: creatinine (cnn), creatine (ctn), citrate (cit), adenosine (ado), indole acetic acid (iaa), 1,3-dimethylurate (dmu), choline (cho).

Judging from the spectra, many acids and other highly polar compounds have been eliminated during extraction, as there are significantly less signals observed between 5.5 and 9.2 ppm. The features of both the extract and the full urine after solvent exchange are qualitatively consistent with the ones previously reported by Reile *et al.*[5]

In addition, a spectrum of urine extract was acquired on an 800 MHz spectrometer, equipped with a He-cooled QCI cryo-probe (Figure S6). General spectral features are consistent with those found in the extract spectrum at 500 MHz shown in Figure S5. The inset shows a zoomed-in view of the NAM region of the spectrum, overlaid with a green trace showing the resonances of NAM. The spectrum displays resonances in addition to those stemming from NAM, which were not identified.

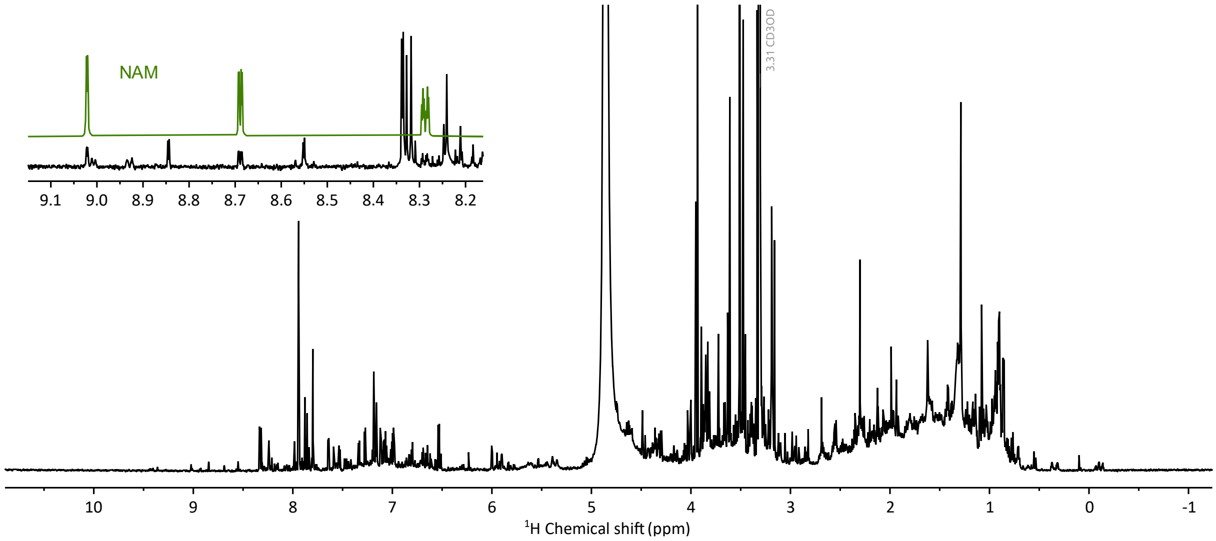

Figure S6. Autoclaved urine SPE extract 1D ^{1}H spectrum measured at 298 K on an 800 MHz spectrometer. A total of 16 scans were collected with acquisition time of 2 s. Data was acquired with 32k points over a spectral width of 16 kHz. Spectra were processed in MestreNova: zero filled to 64k points, exponential line broadening of 0.3 Hz, manually phase- and baseline-corrected using a 3rd order polynomial. Residual methanol-d_4 signal at 3.31 ppm was used as a chemical shift reference.

Finally, a urine extract sample was investigated on a 500 MHz spectrometer using both thermal equilibrium polarization and SABRE. Each spectrum was acquired in a single scan on a solution consisting of extract diluted to 75% and 0.2 mM iridium catalyst. For SABRE, 50% enriched $p\mathrm{H}_2$ was bubbled through the sample for 20 s by a 0.8 mm O.D. PEEK capillary, in a polarization transfer field of 6.5 mT before transfer into the spectrometer.

The higher chemical shift distribution in frequency space allows for a better baseline resolution, despite the linewidth being similar to the benchtop setup at 3 Hz. This makes it possible to identify additional resonances in the aromatic region, which on the benchtop setup overlapped with signals assigned to NAM.

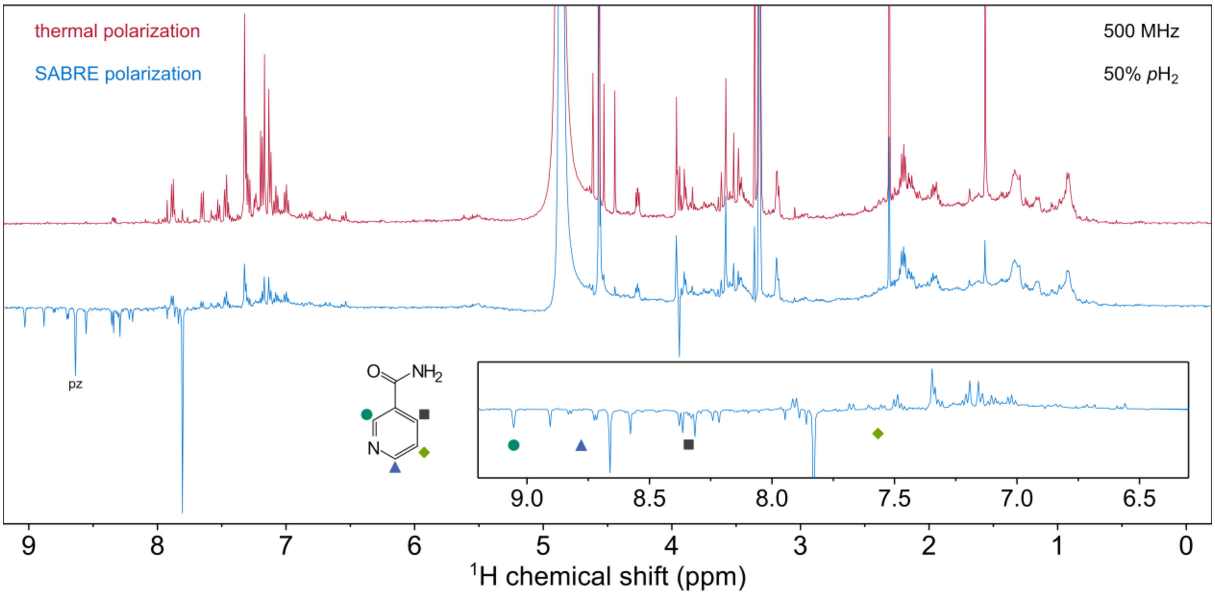

Figure S7. Spectra of Urine extract recorded on a 500 MHz spectrometer using thermal and SABRE polarization. The employed parahydrogen was enriched to 50%. The inset shows a magnified view of the SABRE spectrum's aromatic region, including peaks assigned to NAM protons. Additional signals in this region are clearly separated. The sample contained traces of pyrazine (pz), yielding the marked SABRE signal.

Urine extract investigation by nh-PHIP

The solid phase extract of autoclaved urine was also analyzed by nh-PHIP, according to methods published previously.[6,7] nh-PHIP and SABRE employ the same iridium catalyst, but differ in the nature of the detected signals. In nh-PHIP, analytes are detected by the iridium bound hydride signals of their complexes with the catalyst, rather than by direct enhancement of the analyte resonances. The chemical shifts of these hydrides appear in the -15 to -30 ppm region and are highly sensitive to the nature of the analyte occupying the *trans* position of the hydride in the catalyst complex.

nh-PHIP spectra were recorded at 25 °C sample temperature on an 800 MHz Bruker Avance III spectrometer equipped with a He-cooled cryoprobe. 1D nh-PHIP spectra were recorded with the SEPP pulse sequence, with selective refocusing pulses that cover the whole hydride spectral region, in 16 or 32 scans, utilizing 1.5 sec of 50% enriched pH_2 bubbling between scans. Hyperpolarized 2D spectra were recorded with the 2D nh-PHIP zero quantum (ZQ) experiment with selective pulses, as described by Sellies *et al.*[8] Spectral widths were 8012 and 2000 Hz in f2 and f1 dimensions, respectively. Datasets consisted of 4096 (complex) × 512 (real) points. pH_2 was bubbled through the sample for 2.5 s between scans. A 90° shifted square sine window function was applied in both dimensions, f1 dimension was zero-filled to 1024 points and f2 dimension to 8192 points before Fourier transformation.

The obtained nh-PHIP spectrum (Figure S8) reveals numerous compounds that are able to coordinate to iridium. We were able to identify NAM and adenosine derivatives.

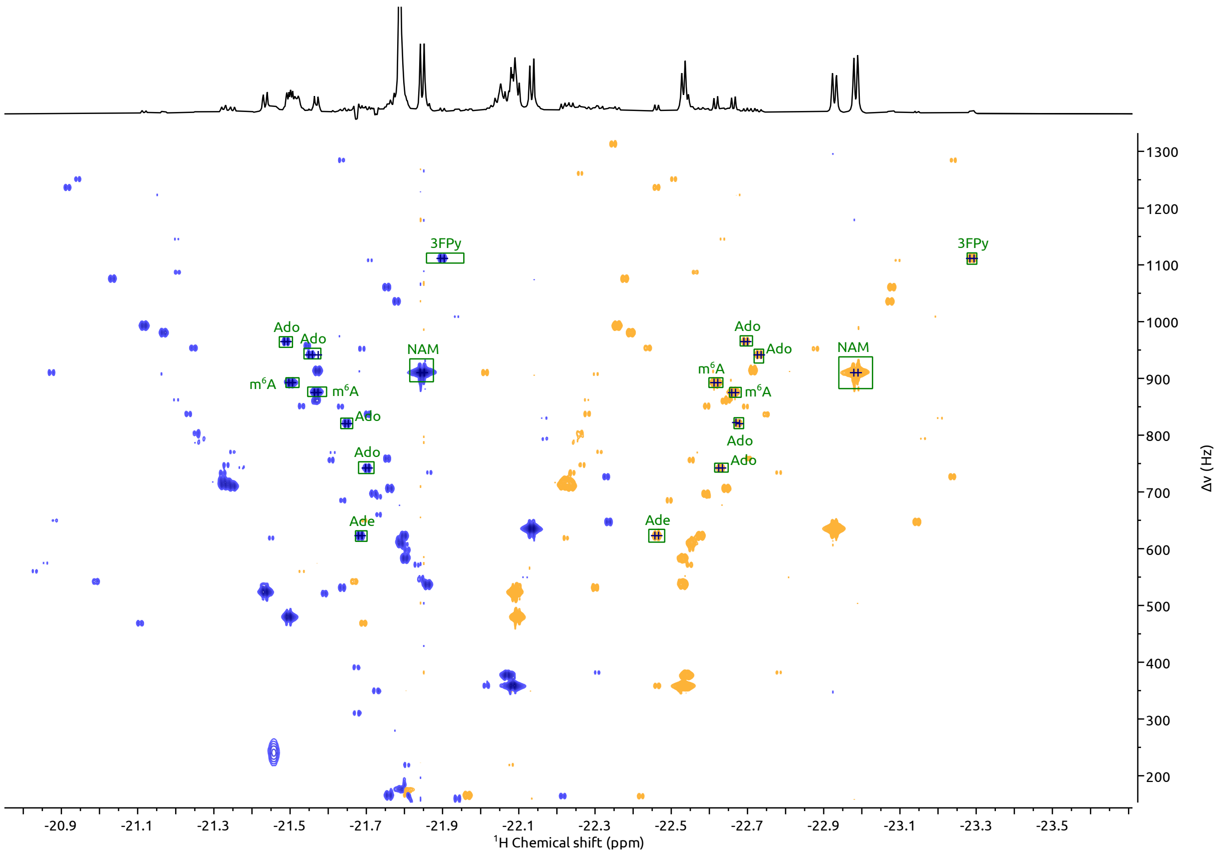

Figure S8. 2D nh-PHIP zero quantum spectrum of urine extract, using 50% enriched pH_2. We identified several compounds: nicotinamide (NAM), adenosine (Ado), N^6-methyladenosine (m^6A) and adenine (Ade). The sample comprised of 1.2 mM of [Ir(IMes)(COD)]Cl, 21.5 mM of 1-methyl-1,2,3-triazole, 8.3 µM of 3-fluoropyridine and 370 µL of urine extract. 3FPy was used as a hydride chemical shift reference.

nh-PHIP signal integrals are dependent on the binding kinetics of the analyte and the iridium catalyst, as is the case for SABRE.[9] Accurate analyte concentration determinations are possible with per-analyte calibration curves. However, analyte concentrations can also be

roughly estimated by comparing analyte nh-PHIP signal integral ratios to a known concentration internal standard (3-fluoropyridine in this work), provided that their relative nh-PHIP efficiency is known (Figure S9).

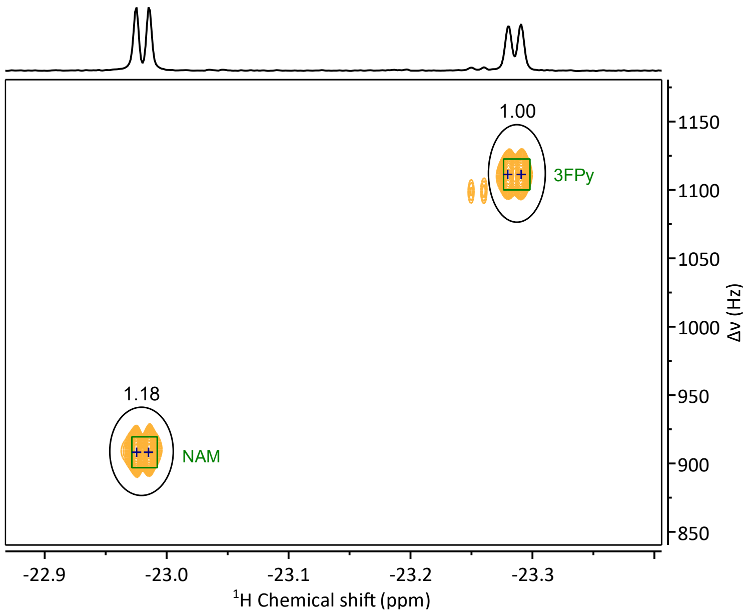

Figure S9. Establishing relative nh-PHIP efficiencies for 3-fluoropyridine (3FPy) and nicotinamide (NAM) complexes. The sample contained equal concentrations of the two analytes (10 µM each) and the otherwise usual nh-PHIP catalyst system of 1.2 mM of [Ir(IMes)(COD)]Cl, 21.5 mM of 1-methyl-1,2,3-triazole. The 2D nh-PHIP zero quantum spectrum was acquired under identical experimental conditions to urine spectra in Figure S8, using 50% enriched pH_2. Comparison of the integrals of the hydride signals of both analytes allowed to establish relative nh-PHIP enhancement factors as 1:1.18 3FPy:NAM. This ratio was used to estimate NAM concentration in the urine (Figure S10).

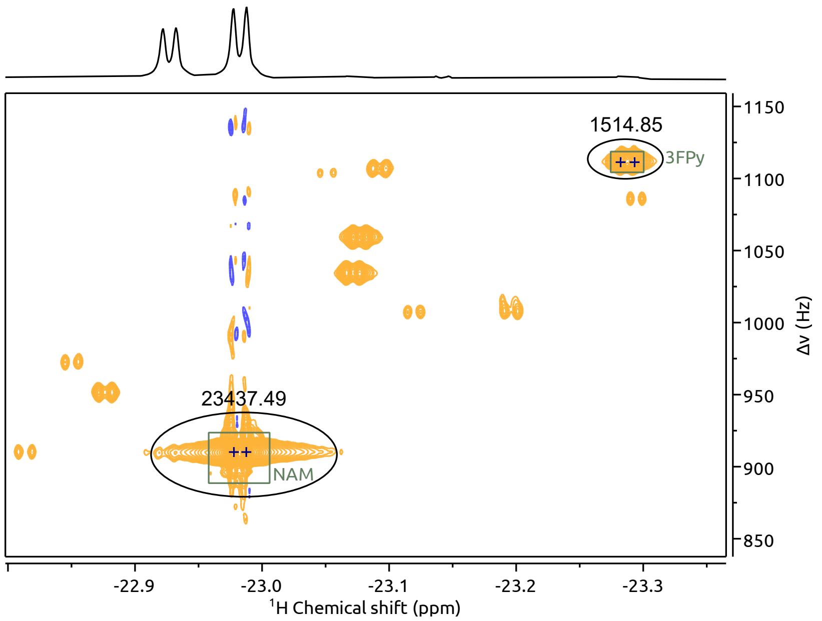

Figure S10. An expansion from Figure S7, focused on the right-hand side hydride signals of 3FPy and NAM complex of the 2D nh-PHIP zero quantum spectrum of urine extract. The absolute integrals of 3FPy and NAM are shown. Integral values allow to estimate the urine extract NAM concentration.

By using the relative concentration determination, we can calculate that NAM concentration based on the added 3FPy (8.3 µM) in the autoclaved urine SPE extract is approx. 128 µM. But

in this case, we have to take into account the relative nh-PHIP efficiencies for NAM and 3FPy (1.18), therefore the concentration of NAM in the NMR tube is approx. 150 µM. The urine extract was diluted 1.6-fold in the nh-PHIP experiment, therefore, the actual urine extract contains approx. 240 µM of NAM. This suggests approx. 40 µM in the initial urine, considering analyte concentration increase during SPE. This value roughly aligns with 51 µM NAM reported in the main text using SABRE.

References

1 I. Reile, N. Eshuis, N. K. J. Hermkens, B. J. A. Van Weerdenburg, M. C. Feiters, F. P. J. T. Rutjes and M. Tessari, *Analyst*, 2016, **141**, 4001–4005.
2 R. Savka and H. Plenio, *Dalton Trans.*, 2015, **44**, 891–893.
3 M. J. Cowley, R. W. Adams, K. D. Atkinson, M. C. R. Cockett, S. B. Duckett, G. G. R. Green, J. A. B. Lohman, R. Kerssebaum, D. Kilgour and R. E. Mewis, *J. Am. Chem. Soc.*, 2011, **133**, 6134–6137.
4 N. Eshuis, B. J. A. van Weerdenburg, M. C. Feiters, F. P. J. T. Rutjes, S. S. Wijmenga and M. Tessari, *Angewandte Chemie*, 2015, **127**, 1501–1504.
5 I. Reile, N. Eshuis, N. K. J. Hermkens, B. J. A. Van Weerdenburg, M. C. Feiters, F. P. J. T. Rutjes and M. Tessari, *Analyst*, 2016, **141**, 4001–4005.
6 N. Reimets, K. Ausmees, S. Vija and I. Reile, *Anal. Chem.*, 2021, **93**, 9480–9485.
7 N. Reimets, K. Ausmees and I. Reile, *Journal of Magnetic Resonance Open*, 2024, **21**, 100171.
8 L. Sellies, I. Reile, R. L. E. G. Aspers, M. C. Feiters, F. P. J. T. Rutjes and M. Tessari, *Chem. Commun.*, 2019, **55**, 7235–7238.
9 K. Lin, P. TomHon, S. Lehmkuhl, R. Laasner, T. Theis and V. Blum, *ChemPhysChem*, 2021, **22**, 1947–1957.
